# Editorial: Community series in primary immunodeficiencies worldwide, volume II

**DOI:** 10.3389/fimmu.2025.1564959

**Published:** 2025-02-19

**Authors:** Antonio Condino-Neto, Anne-Sophie Korganow, Hirokazu Kanegane

**Affiliations:** ^1^ Department of Immunology, Institute of Biomedical Sciences, University of Sao Paulo, Sao Paulo, SP, Brazil; ^2^ Hôpitaux Universitaires de Strasbourg Strasbourg, Strasbourg, France; ^3^ Department of Child Health and Development, Institute of Science Tokyo, Tokyo, Japan

**Keywords:** primary immunodefciencies, inborn errors in immunity, epidemiology, genetic screen, newborn screen (NBS)

Primary immunodeficiencies (PIDs), also referred to as inborn errors of immunity, are rare but impactful conditions characterized by impaired immune function, leading to increased susceptibility to infection and immune dysregulation. This Research Topic summarizes the key findings from the second volume of the Research Topic Community Series, focusing on advances in genetic understanding, diagnostics, and management strategies.

## Expanding the genetic and clinical landscape of PIDs

Research continues to uncover the genetic basis of PIDs, shedding light on their clinical variability. A case report by Chen et al. illustrates how mutations in the recombination-activating gene 1 (*RAG1*) can present atypically as autoimmune hemolytic anemia. By leveraging next-generation sequencing and lymphocyte subset analysis, the case underscores the need for genetic tools to identify atypical PID presentations.

Similarly, Jiang et al. report a novel mutation in the *IL2RG* gene linked to X-linked severe combined immunodeficiency (X-SCID). Despite intensive anti-infective treatment, the patient succumbed to disseminated BCG disease. This case highlights the critical role of early diagnosis and timely allogeneic hematopoietic cell transplantation (HCT), emphasizing the importance of newborn screening (NBS) to improve outcomes for rare PIDs such as X-SCID.

## Newborn screening and early intervention

NBS has transformed the early detection of PIDs, enabling life-saving interventions. Beppu et al. document the successful diagnosis of X-SCID in Japan through NBS, which enabled early HCT without complications. However, the global implementation of NBS faces challenges, such as limited infrastructure and disparities in healthcare access.

Äng et al. demonstrate the limitations of current TREC-based NBS algorithms, which fail to detect certain PIDs, such as *IKZF1*-related combined immunodeficiency. This underscores the importance of integrating kappa-deleting recombination excision circles into NBS protocols to capture a broader range of immune disorders.

## Innovative diagnostic and screening tools

Cost-effective diagnostic tools remain essential in resource-limited settings. Sartorelli de Toledo Piza et al. propose calculated globulin as a viable screening alternative for antibody deficiencies, particularly hypogammaglobulinemia. Their findings suggest that calculated globulin has a strong correlation with IgG levels, thus bridging diagnostic gaps in underserved regions.

Flow cytometry (FCM), as demonstrated by Tahiat et al., offers a versatile and cost-effective diagnostic method for PIDs such as SCID, Omenn syndrome, and familial hemophagocytic lymphohistiocytosis. In addition to identifying hallmark immunodeficiencies, FCM provides clues for conditions such as hyper-IgE syndrome and *STAT1* gain-of-function mutations, highlighting its adaptability in diverse clinical environments.

## Immune dysregulation and malignancy in PIDs

Immune dysregulation, a common feature of PIDs, is associated with a heightened risk of malignancy and organ damage. Graafen et al. explore the immune profiles of patients with ataxia telangiectasia (AT), revealing deficiencies in T- and NK-cell function. These findings suggest therapeutic potential in targeting cytokine pathways or enhancing T- and NK-cell functionality.

Boyarchuk et al. offer a comprehensive review of Nijmegen breakage syndrome, a disorder linked to DNA repair defects, immunodeficiency, and malignancy. Their 25 years of experience in Ukraine underscore the urgency of regular monitoring, early chemotherapy, and immunoglobulin replacement therapy to mitigate risks. Notably, malignancy remains the leading cause of death in Nijmegen breakage syndrome, necessitating advancements in molecular diagnostics and therapeutic interventions.

## Rare and atypical presentations

The phenotypic variability of PIDs presents diagnostic challenges. Al-Saud et al. describe a mild case of *STK4* mutation-related immunodeficiency with severe T-cell lymphopenia, showcasing how functional analysis can delineate disease severity. Similarly, Fink et al. report on severe aplastic anemia associated with *STAT1* gain-of-function mutations, broadening the understanding of immune dysregulation’s impact on hematologic health.

## Future directions

Collectively, the articles included in this Research Topic highlight progress in PID research, particularly in genetic diagnostics, NBS, and cost-effective tools such as calculated globulin and FCM. They also underscore the need to manage immune dysregulation and malignancies in conditions such as AT and Nijmegen breakage syndrome. Expanding NBS programs to underserved regions is crucial for reducing diagnostic delays.

Moreover, advancements in gene therapy and targeted biologics hold promise for transforming PID management. International collaboration will be vital in fostering equitable access to these innovations. For example, Sartorelli de Toledo Piza et al. illustrate how calculated globulin complements traditional IgG assessment, and Tahiat et al. demonstrate the diagnostic versatility of FCM.

## Conclusion

The articles in this Research Topic highlight the complexity and diversity of PIDs, emphasizing the importance of early diagnosis, innovative diagnostic tools, and targeted therapies. By fostering global collaboration and sharing advancements, the PID community can advance the understanding and care for these conditions, ultimately improving outcomes for patients worldwide.

We extend our gratitude to the authors and contributors for their valuable insights and to the readers for their engagement with this Research Topic Community Series. This series plays a critical role in fostering an ongoing dialogue within the PID research community, providing a platform to share advancements, challenges, and innovative approaches to improve patient outcomes globally. Together, we strive for a future where no patient with a primary immunodeficiency is left undiagnosed or untreated.

